# Effect of Different Primers on the Shear Bond Strength of Orthodontic Brackets Bonded to Reinforced Polyetheretherketone (PEEK) Substrate

**DOI:** 10.3390/dj12060188

**Published:** 2024-06-20

**Authors:** Ahmed Akram EL-Awady, Khaled Samy ElHabbak, Hussein Ramadan Mohamed, Ahmed Elsayed Elwan, Karim Sherif Adly, Moamen Ahmed Abdalla, Ehab Mohamed Kamal, Ahmed Leithy Alameldin

**Affiliations:** 1 Department of Orthodontics, Faculty of Dental Medicine (Boys), Al-Azhar University, Cairo 11651, Egypt; 2 Department of Orthodontics, Faculty of Oral and Dental Medicine, MTI University, Cairo 4416301, Egypt; khaled.al-habbak@dnt.mti.edu.eg; 3 Crown and Bridge Department, Faculty of Dental Medicine (Boys), Al-Azhar University, Cairo 11651, Egypt; husseinramadan.209@azhar.edu.eg (H.R.M.); ahmedelwan.209@azhar.edu.eg (A.E.E.); karimadly.209@azhar.edu.eg (K.S.A.); ahmed.alameldin@azhar.edu.eg (A.L.A.); 4 Department of Substitutive Dental Science, College of Dentistry, Imam Abdulrahman Bin Faisal University, Dammam 34212, Saudi Arabia; maabdalla@iau.edu.sa; 5 Operative Dentistry Department, Faculty of Dental Medicine (Boys), Al-Azhar University, Cairo 11651, Egypt; ehabkamal.209@azhar.edu.eg

**Keywords:** shear bond strength, PEEK, orthodontic adhesives, orthodontic primer, metallic brackets, Bio-HPP

## Abstract

This in vitro study assessed the effect of different primers on the shear bond strength (SBS) and adhesive remnant index (ARI) of orthodontic brackets bonded to reinforced polyetheretherketone (PEEK) substrate. A total of 40 specimens were randomly distributed to two groups based on the primer used for orthodontic bonding: group 1 (control)—Transbond XT adhesive with Visio.link primer and group 2 (test)—orthodontic adhesive (Transbond XT) with traditional orthodontic primer. After bonding, specimens were thermocycled followed by SBS testing and ARI scoring of debonded specimens. Data were analyzed using the unpaired independent *t*-test and the Chi-square test. Group 1 specimens showed significantly higher SBS values (21.38 ± 1.48 MPa) compared to group 2 specimens (18.63 ± 1.29 MPa) (*p* < 0.0001). Adhesive remnant index scores showed no significant variations in bond failure modes and distributions between groups. The SBS obtained by the tested primers exceeded the clinically recommended value. Consequently, there is a comparable clinical application for both tested primers in orthodontic bonding, especially the traditional orthodontic primer, where the availability of Visio.link in clinical practice is not ensured.

## 1. Introduction

Bonding orthodontic brackets clinically is of paramount importance for the overall effectiveness of orthodontic treatment [1]. In the past few years, adult orthodontic treatment has gained popularity [1,2,3]. Accordingly, there may be clinical scenarios in which orthodontic brackets are bonded to prosthetic or restorative surfaces. When a fixed dental prosthesis is planned for an orthodontic patient, it is typically recommended that the insertion of the final prosthetic restoration be delayed until the completion of orthodontic therapy [1]. This delay permits the tooth to be repositioned to its final position after orthodontic treatment is completed before placing the final restoration [2,3]. In addition, any surface damage to the fixed restoration upon removal of bonded orthodontic brackets may necessitate replacement of the fixed dental restoration [3]. Therefore, interim restorations are placed between the completion of orthodontic therapy and the placement of the final prosthetic restoration. Furthermore, they prevent tooth decay and fracture while protecting the pulp and periodontal tissues [2,4].

Computer-aided design and computer-aided manufacturing (CAD/CAM) technology have facilitated using pre-polymerized materials to fabricate long-term temporary restorations with lower liability for fracture and marginal discrepancy than the direct method [3]. Polymethylmethacrylate (PMMA) is a widely utilized material for fabricating interim crowns using CAD/CAM technology [5]. However, polyetherether-ketone (PEEK), a high performance polymer (HPP), has emerged as a potential material for indirect long-term interim restoration fabrication in recent years. PEEK is a thermoplastic, aromatic, semi-crystalline material with a linear structure resulting from the binding of ether and ketone groups around aryl rings [6,7]. The growing popularity of PEEK among dental practitioners is due to its low modulus of elasticity, which closely resembles natural bone, high resistance to chemical wear hydrolysis, superior mechanical properties, and high temperature resistance [8,9]. Nevertheless, the inherent hydrophobic nature of unmodified PEEK limits its clinical use, and wetting the material with hydrophobic resin is challenging [10].

Reinforced PEEK is known as bioactive PEEK, and contains ceramic fillers or Bio-HPP, 20% of which contains ceramic fillers [7,8]. The white shade and excellent polishability of Bio-HPP makes it a potential alternative to other restorative materials for the fabrication of fixed dental restorations [8]. Bio-HPP has emerged as an alternative for tooth-colored crowns [10,11], especially for people with parafunctions such as bruxism, because it does not abrade antagonist teeth [7]. It can also be used to fabricate endo-crowns to manage endodontically treated teeth in young adolescents and adults [7].

However, bonding to reinforced PEEK has been an area of interest for recent research [11] in which air-borne particle abrasion or sandblasting using aluminum oxide (Al_2_O_3_) particles with or without silica coating has been proposed as a suitable method for the surface modification of PEEK. This creates micro-roughness for micro-mechanical interlocking with resin and makes it more prone to moisture, allowing for better wetting with more hydrophobic resins [11,12]. Chemical modifications have also been proposed to enhance bonding to PEEK-based materials, including the application of adhesive primers such as Visio.link^®^ (Bredent, Germany) or Signum PEEK Bond^®^ (Kulzer GmbH, Hanau, Germany) [12]. Combined mechanical surface roughening and chemical adhesive treatments are recommended to improve material adhesiveness to a wetting hydrophobic resin by diversifying functional groups and creating a micromechanical interlock [12].

In the literature, combining mechanical surface pretreatment with adhesive primers, namely PEEK’s adhesive primer Visio.link, is proposed to increase shear bond strength (SBS) [12]. As a routine surface treatment, it is recommended that Visio.link be used as the adhesive primer when bonding to PEEK [6,12]. However, in a systematic review of adhesion to HPP in dentistry, Gama et al. [12] concluded that only a few studies had been conducted to compare SBS among specimens using various bonding adhesives, which was insufficient for them to conduct a meta-analysis. Nonetheless, in a study by Lee et al. [13] different dental adhesives and primers were applied to surface-treated PEEK, and it was concluded that a self-etching universal primer containing both MDP and a silane coupling agent is a suitable alternative to the manufacturer’s recommended primer. During orthodontic bonding, Visio.link is often less readily available to orthodontists than orthodontic primers. Nonetheless, the recommendation to use different dental adhesive primers when bonding to PEEK in clinical practice is unclear.

Therefore, the aim of this study was to assess the effect of different primers (the manufacturer’s recommended primer or an orthodontic primer) on the shear bond strength and adhesive remnant index (ARI) of orthodontic brackets bonded to reinforced PEEK. The null hypothesis of the current study was that the SBS of metallic orthodontic brackets bonded to reinforced PEEK using two adhesive primers would not vary significantly.

## 2. Materials and Methods

### 2.1. Specimen Preparation

**Figure 1:**
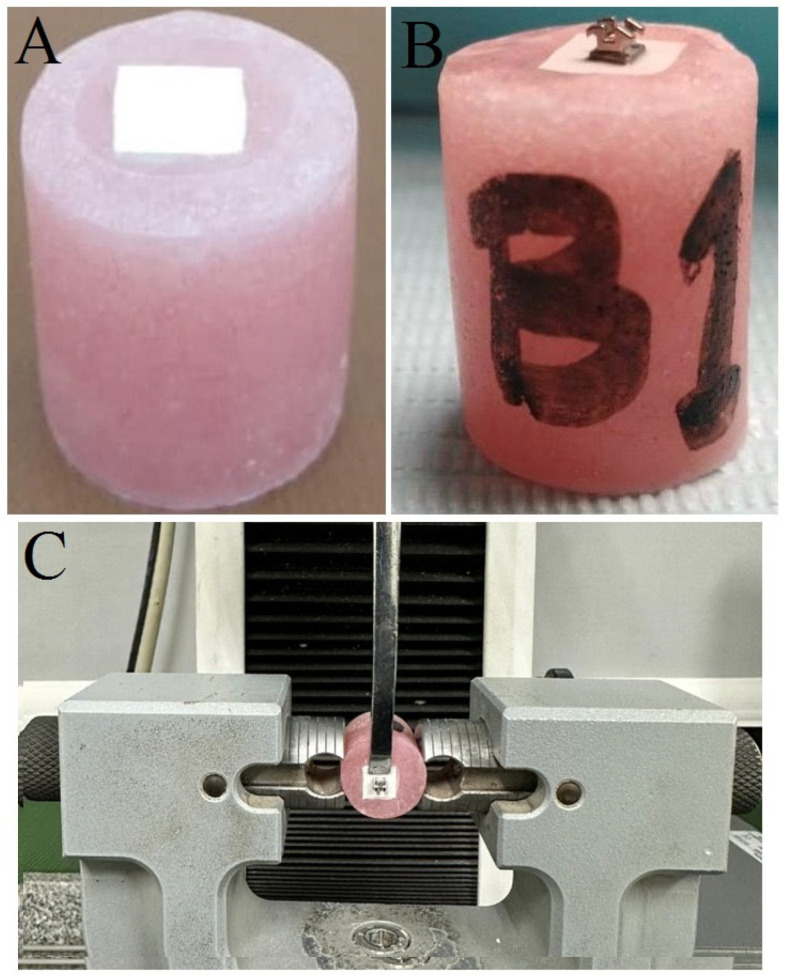
Specimen preparation and SBS set-up. (**A**) PEEK specimens embedded in acrylic; (**B**) orthodontic bracket bonded to surface-treated specimen, and (**C**) specimen mounted in universal testing machine for SBS test.

According to a prior investigation [14], 40 specimens (*n* = 20 for each group) were adequate to show significant differences in SBS between groups. Forty rectangular specimens (5 × 7 × 2 mm^3^) were sectioned from prefabricated PEEK blanks (breCAM, Bio-HPP; Bredent GmbH, Senden, Germany). PEEK blanks were sectioned as per the required dimensions utilizing an automated precision saw (Isomet^®^ 4000, Buehler, Lake Bluff, IL, USA) using a diamond cut-off wheel saw at 2500 rpm and a continuous water coolant [15]. Next, sectioned specimens were contained in individual acrylic cylinders using self-curing acrylic resin (Acrostone Dental and Medical Supplies, Cairo, Egypt) (Figure 1A). The bonding surfaces of all specimens were ground and polished with sequential use of P600 and P800 grit silicon carbide paper (3M™, St. Paul, MN, USA) at 300 rpm under water coolant using an automated LaboPol-25 grinding and polishing machine (LaboPol, Struers GmbH, Copenhagen, Denmark). To standardize the polishing process, all specimens were finished and polished by a single operator (H.R.M). Polished specimens were cleaned for 10 min in an ultrasonic cleaner (Krisbow CD4862, Shenzhen, China) containing distilled water, followed by air-drying prior to surface treatment [5,16,17,18].

### 2.2. Surface Treatment and Specimen Grouping

Surfaces of PEEK specimens were sandblasted using 110 µm Al_2_O_3_ particles for 30 s using a pressure of 0.25 MPa from a distance of 10 mm, followed by 10 min cleaning using distilled water in an ultrasonic unit and air-drying for 30 s [5,17,18,19]. Surface-treated specimens were randomly assigned to one of two groups (*n* = 20) based on the primer application of the bonding surface, as follows:

Group 1 (control): Visio.link (Lot#: 210802, Expiry: 02/24, Bredent GmbH & Co. KG, Senden, Germany).
Group 2 (test): traditional orthodontic primer (Transbond XT, Lot#: MB4GA, Expiry: 05/24, 3M Unitek, Monrovia, CA, USA).

### 2.3. Bracket Bonding

Group 1 (control): A thin film of Visio.link primer was applied onto the surface-treated PEEK surface, distributed via gentle air spray from a distance of 15 mm, and light-cured for 90 s using a hand-held unit (1000 mW/cm^2^, Woodpecker Guilin, Guangxi, China) [17]. Metal mandibular incisor brackets (0.022-inch, Gemini series, 3M Unitek, Monrovia, CA, USA) with a 10.5 mm^2^ average bonding area were bonded to the PEEK surface. The bracket base was covered with a small amount of orthodontic adhesive (Transbond XT, Lot#: MB4GA, Expiry: 05/24, 3M Unitek, Monrovia, CA, USA) and pressed against the specimen’s surface. Excess adhesive was removed (Figure 1B) and bracket surfaces were light-cured for 40 s [20,21].

Group 2 (test): The treated surface was coated with a thin film of Transbond XT primer, distributed onto the specimen surface via gentle air spray at a distance of 15 mm, and light-cured for 10 s. Bracket bonding was identical to the procedure detailed for group 1 specimens.

Bonded specimens were thermocycled to replicate the oral environment after being stored in distilled water at 37 °C for 24 h. The thermocycler apparatus (SD Mechatronik, Feldkirchen-Westerham, Germany) operated for 1500 cycles, between 5 to 55 °C, with a dwell time of 20 s and a transfer time of 10 s [22,23,24].

### 2.4. Shear Bond Strength Testing

A random number was allotted to each specimen before testing to avoid bias during testing. SBS tests were performed by a universal testing machine (Model 2710-113, Instron, Norwood, MA, USA). A vertical shear load was applied to each bracket’s base parallel to the PEEK/adhesive/bracket interface at a crosshead speed of 1 ± 0.1 mm/min until fracture (Figure 1C) [25,26,27]. The debonding load was recorded in Newtons (N) and then divided by the bracket base area (10.5 mm^2^) to obtain the SBS in megapascals (MPa) [28].

### 2.5. Adhesive Remnant Index (ARI)

Specimens’ surfaces after debonding were examined utilizing a stereomicroscope under ×20 magnification (Nikon SMZ745T, Tokyo, Japan) to quantify the remaining adhesive amount on the surface. Each debonded specimen was assigned a score based on the adhesive remnant index (ARI), as detailed below [29]:

Score 0 = no Transbond XT adhesive left on the PEEK surface;

Score 1 ≤ 50% of the Transbond XT adhesive left on the PEEK surface;

Score 2 ≥ 50% of the Transbond XT adhesive left on the PEEK surface;

Score 3 = all of the Transbond XT adhesive left on the PEEK surface.

### 2.6. Scanning Electron Microscopy (SEM) Analysis

A representative PEEK substrate was examined under SEM (TESCAN VEGA, Brno-Kohoutovice, Czech Republic) to evaluate sandblasting’s effects on the PEEK surface. Furthermore, a representative specimen was also examined under SEM after debonding to examine adhesive remains on the PEEK surface.

### 2.7. Statistical Analysis

Data analysis was performed with a statistical software package (v.24, IBM SPSS, Armonk, NY, USA). Descriptive statistics were generated for the studied groups. The unpaired independent *t*-test was applied to compare groups, preceded by assessment of normality through Shapiro–Wilk and Kolmogorov–Smirnov tests. These test results indicated a normal distribution of the parametric data under consideration. The Chi-square test was used to compare ARI scores between the studied groups (α ≤ 0.05).

## 3. Results

### 3.1. Shear Bond Strength

Table 1 presents descriptive statistics of the studied groups. The SBS of group 1 (Visio.link primer) ranged between 18.70 MPa and 23.50 MPa. On the contrary, the SBS of group 2 (Transbond XT traditional primer) ranged between 16.75 MPa and 21.10 MPa. The 95% confidence intervals show the range of reasonable values for the true mean SBS in the underlying population, ranging from 20.68 to 22.07 for group 1 and from 18.03 to 19.24 for group 2. Overall, the descriptive statistics indicate that group 1 specimens resulted in higher SBS on average, with more variability, compared to group 2 specimens.

The mean ± SD of SBS values of the studied groups are presented in Figure 1. The mean SBS of group 1 PEEK specimens was 21.38 ± 1.48 compared to 18.63 ± 1.29 for group 2 PEEK specimens.

Table 2 presents results of an unpaired *t*-test comparing the mean SBS between the two groups. With a *p*-value less than 0.0001, the difference in mean SBS was statistically significant. The two-tailed *p*-value indicates this was a non-directional hypothesis test. The large *t*-value of 6.237 with 38 degrees of freedom also reflects the highly significant difference between groups.

### 3.2. Adhesive Remnant Index (ARI)

Table 3 presents a comparative analysis of ARI scores between groups using a Chi-square test. In group 1, there were no specimens with a score of 0, 3 specimens (15%) scored 1, 11 specimens (55%) scored 2, and 6 specimens (30%) scored 3. For group 2, there were no specimens with an ARI score of 0, 5 specimens (25%) scored 1, 10 specimens (50%) scored 2, and 5 specimens (25%) scored 3. Chi-square *p*-values comparing the distributions of scores 1, 2, and 3 individually between the two groups were not statistically significant. This indicates a non-significant difference in the bond failure modes and distribution of ARI scores of Visio.link primer versus Transbond XT orthodontic primer.

### 3.3. Scanning Electron Microscopy (SEM) Observation

**Figure 2:**
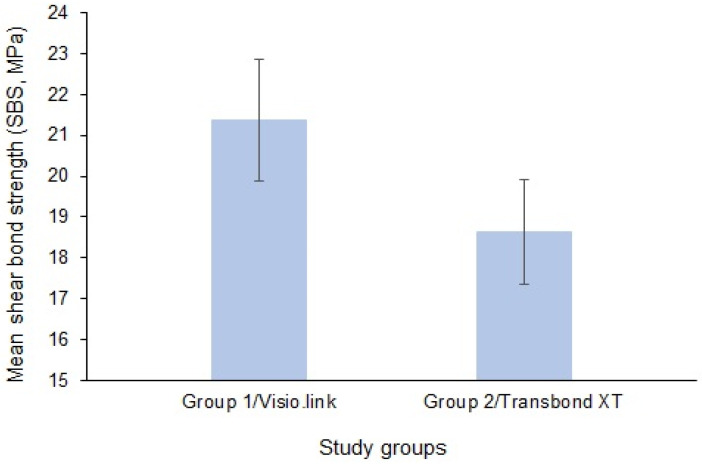
Shear bond strengths of the studied groups.

**Figure 3:**
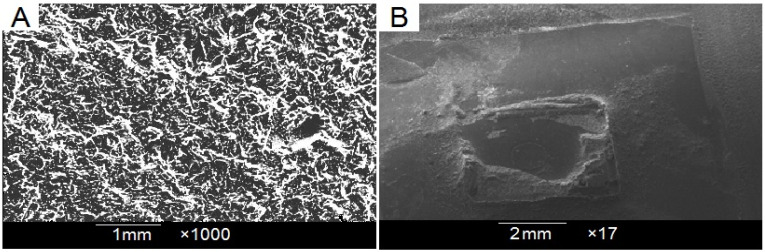
(**A**) Surface-treated PEEK specimen; (**B**) PEEK surface after debonding showing significant amount of remaining adhesive material.

A SEM micrograph of the surface-treated and debonded PEEK specimen is presented in Figure 2. The surface-treated PEEK surface (Figure 3A) showed an irregularly speckled pattern with deep grooves and craters. The PEEK specimen, after debonding (Figure 3B), showed a significant amount of adhesive remaining on most bracket base surface areas (representing an ARI score > 2).

## 4. Discussion

The current study assessed the SBS of orthodontic brackets bonded to PEEK surfaces using either manufacturer-recommended adhesive primer or traditional orthodontic primer. To the best of the authors’ knowledge, this is the first study to assess the SBS of orthodontic brackets bonded to reinforced PEEK. It was hypothesized that the SBS of orthodontic brackets bonded to reinforced PEEK using two adhesive primers would not significantly vary. The data analysis outcome recommends rejecting the hypothesis, as there was a significant difference in SBS values between the two studied groups.

The literature recommends that the minimum SBS be in the range of 6–8 MPa, and the maximum SBS must be lower than the fracture threshold of enamel, which is around 14 MPa [23,30]. However, it must be remembered that SBS obtained in vitro are typically 40% higher than those obtained in vivo owing to the complexity of the oral environment, where moisture contamination can noticeably reduce the SBS [31]. The current study demonstrated that the SBS of brackets bonded using Visio.link primer and traditional orthodontic primers were 21.38 ± 1.486 MPa and 18.63 ± 1.291 MPa, respectively. The difference in SBS between the primer groups was statistically significant; however, the difference seemed clinically irrelevant, as both these values exceeded the recommended optimal SBS values. In orthodontic practice, obtaining optimal SBS rather than the maximum possible SBS is preferable [32]. Brackets bonded to fixed prosthodontic surfaces should be compatible to resist the oral biomechanics, masticatory forces, and functional activities of the patient, but also adequate enough to facilitate debonding upon treatment completion without any deleterious effect on the bonded surface of the restoration [33].

In this study, PEEK specimens were surface-treated using sandblasting, as it is a simple and safe intraoral method for surface pretreatment that increases the micro-roughness and bonding area of PEEK material, subsequently improving the micromechanical interlocking of bonding agents. Additionally, it eliminates all organic contaminants from the material’s surface with surface activation [16,33]. Visio.link adhesive primer is the recommended standard primer for bonding to PEEK surfaces, as it provides a reliable bond between PEEK and resin regardless of the surface treatment [16,17]. However, this primer may not be readily available, and an orthodontist may be compelled to use traditional orthodontic primer. Although different primers were used, Transbond XT adhesive was used to bond the brackets in both groups, as it is regarded as the gold standard light-cured orthodontic adhesive [21]. Previous studies have predominantly used premolar brackets in their SBS studies; however, in the current study, mandibular incisors metal brackets were used, as their flat base provided maximum adaptation to the flat surface of PEEK specimens [34].

The oral environment undergoes temperature fluctuations (between 0 and 65 °C) that can impact the bond strength of adhesives and restorations. The International Organization for Standardization (ISO) recommends a minimum of 500 cycles at 5–55 °C, 20 s of dwell time, and 5–10 s transfer time to simulate an oral environment [35]. These 500 cycles, which mimic less than two months, are insufficient compared to the average orthodontic treatment time, which extends over one year. Conversely, specimens in this study underwent 1500 thermocycles at 5–55 °C to more accurately replicate oral and clinical thermal stress conditions.

The SBS test is frequently employed to assess the adhesion of dental materials. Conventional shear tests are preferred because they are easy to perform, require minimal tools and specimen preparation, and provide an extensive view of the adhesion strength—despite the SBS test being debatable, deemed unsatisfactory, and requiring that many variables be considered [35].

The adhesion remnant index (ARI) is the most popular and simplest method for assessing adhesion between the bonding substrate and bracket base. This qualitative and subjective index is used to evaluate adhesion quality by assessing adhesive remnants on the bonded surface and identifying the bond failure site after bracket removal [31,32,36]. It is a quick and easy procedure requiring no additional apparatus. Previous studies have shown no significant differences in ARI scores between qualitative visual scoring, elemental mapping, and SEM analysis [37,38]. On the contrary, the reliability of how magnification affects adhesive remnant interpretation has been questioned [39].

Surface damage or fracture at the interface may result from a robust adhesion between the bonding surface and the adhesive resin, which is indicated by an increased ARI score. Consequently, to lower the possibility of restoration damage or surface cracking during bracket removal, failure within the adhesive layer or between the bracket base and adhesive is desirable [31,40,41,42]. The current investigation showed a non-significant difference in bond failure types. Bond failures occurred frequently at the bracket–adhesive contact in both groups, leaving nearly all adhesive on the specimen surface (scores 2 and 3) (Figure 2B). Previous studies have indicated an association between failure mode and bond strength, with higher bond strengths associated with more mixed fractures. As indicated by scores 2 and 3, the bracket–adhesive interface can be considered the most advantageous failure site for safe debonding because most of the adhesive remains on the bonding surface [43]. However, the surface should be meticulously finished and polished to remove adhesive remnants.

The present study had a few limitations that are worth mentioning. Although care was taken to simulate the intra-oral environment as closely as possible, factors such as the presence of saliva, occlusal forces, diet, and oral hygiene methods were not considered. Secondly, the significant difference in SBS between primer groups was inconsistent with non-significant ARI values. Finally, the findings of this study could not be compared to other similar studies, as there were no data available on orthodontic bonding to PEEK surfaces at the time of this investigation. Future studies should focus on SBS in terms of PEEK crowns, aesthetic brackets, and different surface conditioning methods. Also, it would be compelling to evaluate the immediate SBS rather than the 24 h SBS, as archwires are ligated to brackets within 15 min of orthodontic bonding in clinical conditions.

## 5. Conclusions

Within this study’s limitations, the following conclusions were drawn from this laboratory study:

(a) SBS values obtained with the use of both tested primers exceeded the clinically recommended value (6–8 MPa).
(b) SBS values of Visio.link primer (21.38 ± 1.48) were statistically significant compared to those of traditional Transbond XT primer (18.63 ± 1.29) (*p* < 0.0001).
(c) There was a non-significant difference in bond failure modes and the distribution of ARI scores between Visio.link and Transbond XT primers.
(d) There is a comparable clinical application for both tested primers in the orthodontic bonding of metal brackets to a PEEK substrate.
(e) The use of readily available traditional orthodontic primer in clinical orthodontic bonding to a PEEK surface is practical if there is no Visio.link available.

## Figures and Tables

**Table 1:** Descriptive statistics of SBS values of the studied groups.

	Group 1	Group 2
N	20 specimens	20 specimens
Minimum	18.70 MPa	16.75 MPa
25% Percentile	19.84 MPa	17.54 MPa
Median	21.84 MPa	18.52 MPa
75% Percentile	22.36 MPa	19.30 MPa
Maximum	23.50 MPa	21.10 MPa
Mean	21.38 MPa	18.63 MPa
Standard Deviation	1.48 MPa	1.29 MPa
Standard Error of Mean	0.33 MPa	0.28 MPa
Lower 95% Confidence Interval	20.68 MPa	18.03 MPa
Upper 95% Confidence Interval	22.07 MPa	19.24 MPa

**Table 2:** Comparative statistics of SBS between groups using unpaired independent *t*-test.

**Unpaired Independent *t*-Test**	
*p* value	<0.0001
Significant difference (*p* < 0.05)	Yes
One- or two-tailed *p* value?	Two-tailed
*t*, df	*t* = 6.237, df = 38

**Table 3:** Comparative statistics of adhesive remnant index (ARI) scores between groups using Chi-square test.

	ARI 0	ARI 1	ARI 2	ARI 3	Total	*p*-Value
Group 1	0	(3) 15%	(11) 55%	(6) 30%	20	0.6385 (NS)
Group 2	0	(5) 25%	(10) 50%	(5) 25%	20	
*p*-value		0.4350 (NS)	0.7546 (NS)	0.7266 (NS)		

Please refer to text for description of ARI scores; NS—non-significant difference.

## Data Availability

Available data are contained within the article.
